# User Experience of the Mobile Terminal Customization System: The Influence of Interface Design and Educational Background on Personalized Customization

**DOI:** 10.3390/s21072428

**Published:** 2021-04-01

**Authors:** Minzhe Yi, Ying Wang, Xiaoxue Tian, Huichao Xia

**Affiliations:** 1 School of Art and Design, Zhejiang Sci-Tech University, Hangzhou 310018, China; winered@zstu.edu.cn (Y.W.); xiaoxue_tian@foxmail.com (X.T.); xhc00000@zstu.edu.cn (H.X.); 2 Institute of Zhejiang Sci-Tech University-Ouhai, Wenzhou 325000, China

**Keywords:** customization system, interface design, personalized customization, user experience

## Abstract

The study verified the role that different interface designs and users’ educational backgrounds play in the task performance and subjective evaluation of mobile terminal customization system. Interface type (based on scroll, alternative, and attribute) and user group (college students and industrial workers) were employed as the variables. A total of 72 users were included in the study, and an analysis of 3 × 2 between-participants design indicated that (1) Different interface designs of customization systems had a significant difference in task performance, the alternative based interface had the best results in the task performance, and there was no significant difference between the attribute-based and scroll-based interfaces in task performance; (2) The matching between educational background and interface type will affect the users’ evaluation on system usability. Industrial workers thought that the scroll-based and alternative-based interfaces were more useable, while college students preferred attribute-based interface design; (3) Different interfaces had a significant difference in user task load. The scroll-based interface had the lowest mental demand on the users, while alternative-based had the lowest physical demand on the users, though it consumed more effort; (4) Different educational backgrounds had a significant difference in user task load. Industrial workers showed lower effort in the scroll-based and alternative-based interfaces, while college students had lower effort in the attribution-based interface; (5) A correlation analysis showed that there was a significant negative correlation between the system usability score and the effort in task load. This study results have a positive significance for interface design. With educational background and layout as two important factors in our interface design, we may obtain the most appropriate design principles for enhancing the online customization experiences of different groups of consumers. The more important is that this study is based on the actual needs of the industry. For the first time, we take suitcase as an online customized product, which may not only help local manufacturers to extend their traditional offline distribution channels to online, but also provide a constructive thinking concerning interface design for customization of a single product.

## 1. Introduction

Technological progress and social development make people’s personalized demand for products and services increasingly prominent. Against such a backdrop, product customization system is bringing excess profits and competitive advantages to enterprises and gradually becoming their core competitiveness. At the beginning of the 21st century, consumers were not keen on mass customization. However, with the increase of purchasing power of millennials and Gen Z consumers, people who are interested in expressing individuality through products and tend to show their daily life on social media begin to pay great attention to online customization [1]. In online product customization, customers interactively select various design elements of industrial products, indicating that assisting users to make correct choices effectively is the key to the interface design of customized systems [2,3,4].

An important theoretical basis for product customization system is the Theory of Cognitive Fit, which explores how to optimize the match between visual form of information and decision-making task. Vessey and Galletta [5], founders of the theory, pointed out that task performance can be improved when an information presentation form matches a task correctly. This theory has been verified in many industries, such as open idea sourcing [6], consumer web behavior [7], complex managerial decisions [8], and software comprehension [9]. This theory is also effective in customization systems design because the information presentation mode of the interface is closely related to the performance and subjective feeling of the customization task. Kang and Lee [10] pointed out that perceived usability in a customization system is more important than perceived enjoyment in determining the willingness to customize, and they held that the system should allow users to match their favorite interfaces more freely.

**Figure 1:**
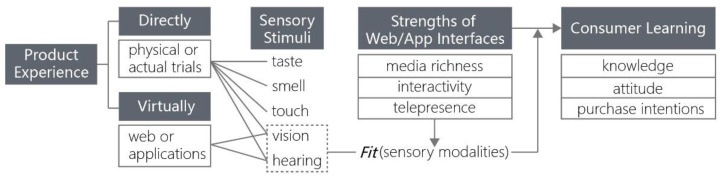
Vision and hearing cues fitting for sensory modalities of Human Machine Interface (HMI) are helpful for the consumers learning about products.

In the product customization system, there is also a function of virtual experience for products, in which the theory of cognitive fit is applied as shown in Figure 1. The virtual experience cannot satisfy all of the five human sensory stimuli covered by direct experience, and so, it transmits product information to the consumers mainly through visual and auditory senses. However, the interfaces of Web and App enable the consumers to learn about products for their advantages in media richness, interactivity, and telepresence, especially when the sensory modalities adopted by Human Machine Interface (HMI) can fit the vision and hearing cues that consumers need, the interfaces will be of more significant utility in product presentation [11].

A product customization system is often considered as “space of products” [12] or “space of solutions” [13], which means that interface design should match user preferences and goals, as well as the functionality and aesthetics of the products [14]. More macroscopically, the product customization system, as a Decision Support System, has become an indispensable part of online shopping. The cognition-driven Decision Support System can help the operators make reasonable decisions from human cognition perspective, e.g., through sensing, comprehending, and projecting [15]. According to Kamis et al. [16], since there is no optimal solution for online shopping, it is critical to understand the users’ views and attitudes towards the operating system for the success rate of shopping. Although the Decision Support System has been used to study several aspects of online shopping [17,18,19], this study focused on the level of product customization in the category of interface visual design. For example, Timberland Company showed users color options in each customizable part for the shoes, and the users could try repeatedly until they felt they had created satisfying shoes.

This study is a continuation of Kamis et al. [16] based on the theory of cognitive fit. The investigators discussed the interface of product customization system on desktop computers and proposed that the attribute-based customization method was superior to the alternative-based one in perceived usability and perceived pleasure. There are two reasons for this study to continue their investigation. First, this study was to verify the applicability of these findings on mobile devices. Although computers equipped with larger screens are considered suitable for tasks with higher cognitive intensity [20], mobile terminals are becoming indispensable for people’s life and work with the arrival of mobile internet era. Many scholars have pointed out that there are many differences in operation modes between mobile terminals and desktop computers. Although some interface design rules for desktop computers are applicable to mobile terminals (such as providing feedback mechanism), more than half of the rules are not [21], for the mobile terminals allow users to swipe, zoom, click, and move with fingers, and transmit relevant information more detailed than a static 2D image, which may increase the sensory depth [22,23]. Furthermore, different from desktops fixed in the office or at home, the mobile terminals are used in complex scenarios, and many factors, such as brightness, noise, and weather, may compete for users’ attention [21]. In fact, the differences in hardware and interactive modes did lead to the differences between desktop and mobile terminal in cognitive load [24], search performance [25] and decision-making behavior [26,27].

Second, we noted that Kamis et al. [16] did not take into account the educational background of users. Numerous studies have shown that the difference in education level can affect people’s performance in various cognitive skills. For example, users with low literacy rate may find it difficult to operate the interface without text for the hierarchical information structure of system [28]. Previous behavioral studies have also shown that educational level may affect the performance on test tasks that are often used in the field of neuropsychology [29,30,31]. Lightner [32] also showed that education level affected the users’ visual preference for shopping interface design. Complex interface background and animation did not affect the purchase behavior of highly educated users, while users of lower education level were more interested in design elements that can affect their senses. It should be noted that we do not want to be misconstrued as "blaming the victims" for lack of certain cognitive skills. Instead, we aim to provide support for the vulnerable group.

To sum up, this study is to investigate the influence of different interface types and educational backgrounds on the usage of mobile terminal customization system in terms of visual sensing. We do not aim to eliminate or stick to a certain interface design but aim to understand the advantages of each interface design and know how to enhance the user experience in online customization as a whole. Our study is based on the actual demands of enterprises. The manufacturers of suitcases in Wenzhou City, Zhejiang Province, an important base for suitcases industry in China, want to get rid of the dependence on the single path of OEM (Original Equipment Manufacturer) for profit, and establish direct communication channels with consumers to build brand value through online customization. Therefore, the prototype of customization system established by this study is based on suitcases. The study results have contributed to the online customization theory and practice, expanded the specific application strategy of the theory of cognitive fit in product customization system through an exploratory study on target users in real scenes, and revealed the relationship between education level and interface design type, and relevant influence on user customization process as well.

## 2. Related Work

### 2.1. Background and Trend of Personalized Customization

It has become a common practice for many enterprises to manufacture products precisely according to the specific requirements of customers. Mass customization has brought these enterprises excess profits and competitive advantages, and even become the core competitiveness of some enterprises. Online customization is popular among young people because they attach great importance to aesthetic factors of products, and want functional features and personalities different from mass-produced products through customization [1]. Organic combination with mobile digital terminal is becoming a trend of personalized customization. Arrighi et al. [33] explored an online customized VR design tool for user participation, which allowed users to modify a product prototype directly on a 3D view without having to prototype the product at an early stage of design, thus promoting collaboration between users and designers. Bachvarov [34] realized the users’ participation in product customization through HMD. In a virtual environment, the users can observe, move, and change the properties (such as color, form, label, etc.) of the products in any direction. An advantage of such a customization mode is that a real environment for use of the products may be simulated. What needs to be solved is that some users exposed to the virtual environment for a long time may get headaches, nausea, fatigue, etc. In addition, the resolution of headsets may also affect the users’ customization experience. For personalized customization, another trend is to focus on the influence of users’ personality characteristics on the customization process. For example, Schlager et al. [35] studied whether consumers in different countries would be influenced by others in personalized customization or not, and the results showed that consumers from a country with a holistic thinking style (Japan) are more receptive to suggestions than consumers from a country with an analytical thinking style (Germany). Deselnicu et al. [36] noted physical functions of the elderly and their special requirements for customization of footwear products.

### 2.2. Integration of Decision Support System and Personalized Customization

The combination of decision support system and personalized customization has attracted wide attention. Kamis et al. [16] found that the attribute-based customization method was superior to the alternative-based one in perceived usability and perceived pleasure through a companion. Sandrin et al. [18] studied the online customization systems for automobiles and laptops, and proposed strategies to enhance the users’ uniqueness and self-expression in perception for customization systems. Kang and Lee [10] explored how customization behaviors can enhance the users’ sense of self-efficacy and pointed out that system developers may provide well-designed customization functions in the interface to promote the users’ confidence in customization tasks. Inconsistency between the users’ intents and the design proposals is easy to occur for it is difficult for the users to accurately express their personal preferences. For this, Zeng et al. [37] proposed a "text-image-symbol" spatial mapping strategy and a clustering strategy to reduce the ambiguity effect of users in the process of product customization. Zhou and He [38] used the fuzzy hierarchical model to identify and classify user demands and developed a relevant importance model, by which the classification results for user demands may be better judged, thus enhancing the efficiency of customer customization. It should be noted that according to Godek and Eveland [39], in some cases, personalized customization will neither allow users to achieve a high level of preference matching, nor increase their cognitive ability of decision control. In contrast, when alternatives are offered in a form other than customization, the users may see a wider variety of products. This study has enlightened us that in an online display, if products are presented according to their attributes, the users may underestimate the varieties of products that the company can offer though they are more accessible and organized visually, for many consumers prefer online stores with abundant species, even if the prices are higher than that in other stores.

### 2.3. User Decision Study Based on the Theory of Cognitive Fit

The theory of cognitive fit holds that the ability to solve problems depends on the information format and the nature of the task, and when the information format matches the task, it contributes to the quality and speed for task completion, otherwise it may compromise the decision-making speed and performance of users [4,5]. Gillespie et al. [40] studied the strategies for displaying advertisement products in narrative scenes (such as movies or TV shows) in terms of position and pointed out that only when the positions of products are consistent with the narrative structure of the story (cognitive fit) and emotional tone (emotional fit), can consumers’ positive attitude towards brands be created. Bacic and Henry [41] found that relationship between cognitive effort and traditional decision performance measurement might not be as direct as the theory of cognitive fit suggests. For example, in the symbol recognition task, the recognition accuracy of the participants did differ significantly due to the different symbol information presentation formats, but it did not cause the difference in cognitive effort of the participants. Gichoya et al. [42] applied the theory of cognitive fit to the evaluation on the patients’ understanding of medical images and proposed that CT images consistent with patients’ cognitive ability could help the patients to understand their diseases. If relevant guidance and basic training could be provided in the early stage, the patients’ understanding ability for medical images might be further improved.

**Figure 2:**
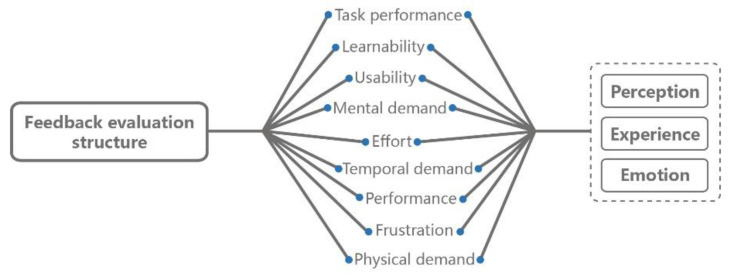
The evaluation framework associated with the customization experience in this study.

To further investigate whether the information presentation of the product customization system is consistent with consumers’ cognitive ability, this study tested the performance of the participants in completing the product customization tasks, and carried out a psychological assessment on the participants at multiple dimensions with Likert scale, so as to comprehensively measure the customization experience of participants based on different interface designs, in which learnability and usability have been widely used to evaluate the effectiveness, safety and enjoyment in interface interactions [43,44]. For relevant evaluation framework, see Figure 2.

## 3. Materials and Methods

**Figure 3:**
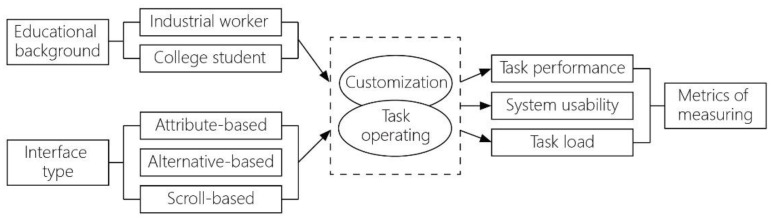
The research model of this study.

The experiment is based on the theory of cognitive fit and the study of Kamis et al. [16], who analyzed two (alternative-based and attribute-based) interface designs of product customization system used for desktop computers and pointed out that the attribute-based interface was superior to the alternative-based one in perceived usability and perceived pleasure. By June 2020, the number of Chinese netizens had reached 940 million, and 99.2% of them use mobile phones to access the Internet, exceeding the proportion of desktop computers (37.3%) and laptops (31.8%) [45], which indicated the importance and urgency of interface design for mobile terminal. The study adopted a 2 (educational background) ×3 (interface type) between-participants design (Figure 3), in which college students and the industrial workers constitute two different types of educational background, and there are three interface designs, i.e., alternative-based, attribute-based, and scroll-based (added in this study) interface designs, for which we will explain in detail in Section 3.1 and Section 3.2. The dependent variables for the experiment are task performance, system usability, and task load scores of users.

### 3.1. Participants

In China, college students are one of the main user groups of suitcases. They are generally resident students and need suitcases for shipping the necessities of life after admission. Another user group of suitcases is urban industrial workers, most of whom are young and middle-aged labor force from rural areas. As the income of urban workers is higher than that of rural workers, a large number of rural workers go to cities for work, and the instability of their working places results in a rigid demand for suitcases. The two user groups are significantly different in educational background. The vast majority of industrial workers have not received high school education, and those with junior high school education level account for 56% of them [46]. This is one of the main reasons why the study includes educational background as a factor.

In this study, the subjects were recruited through a purposive sampling method. Some college students and industrial workers with at least 1 year of experience in using smart phones for online shopping were selected. At last, 72 subjects (43 males, 29 females) aged between 18 and 35 years were included. Among them, 36 are college students and 36 are industrial workers. The college students are the second and third year students of a local university, who have received general education. Industrial workers are recruited from local labor markets. They have not received high school education or education above the level, but they still have basic reading ability and can understand the meaning of various words in the mobile terminal customization system clearly. Nearly 2/3 of the college students uses iPhone, while the remaining 1/3 uses phones of Android system. Only 5 of the industrial workers uses iPhone, while the remaining uses phones of Android system. In order to avoid the influence of different system experiences on the experiment results, the customized system interface did not use the navigation bar or physical buttons on the phones (main differences between android and iOS), but used specially designed “Back” and “Next” buttons to switch optional attributes for the suitcases. In addition, the phones used in the experiment were assigned by the investigators, and the customized system had been installed in the mobile phones before the experiment.

### 3.2. Apparatus and Prototype

The study used a “MockingBot” for interactive prototyping (a popular interface prototyping tool in China), and “Photoshop” and “Illustrator” for image processing, aiming to simulate the product customization system. The interactive prototype was developed for the iPhone 11 Pro Max, with a 6.5-inch screen, 1242 × 2688 resolution, and 458 pixel density. The simulation interface developed through “MockingBot” may run on a mobile phone, and so, the subjects operated using real phones rather than computers.

As for the test interface types, the study redesigned the attribute-based and alternative-based customization interfaces according to the characteristics of the mobile device and supplemented another common interface (we call it as “scroll based” interface). The scroll-based interface was added because it is a common way to browse goods for an online shopping platform, in which consumers may switch the styles of products through clicking the left and right buttons in the interface.

In order to minimize the irrelevant differences between interfaces for this study, one column layout of information structure was adopted for all three interfaces. This layout is suitable for interfaces with little information and simple functions, for it may concentrate information for presentation. Above the interface is the space for attributes, with text for optional attributes of suitcases. In the middle is the space for products, with real time images of suitcases. At the bottom is the space for navigation, with a navigation bar for switching back and forth between custom attributes.

**Figure 4:**
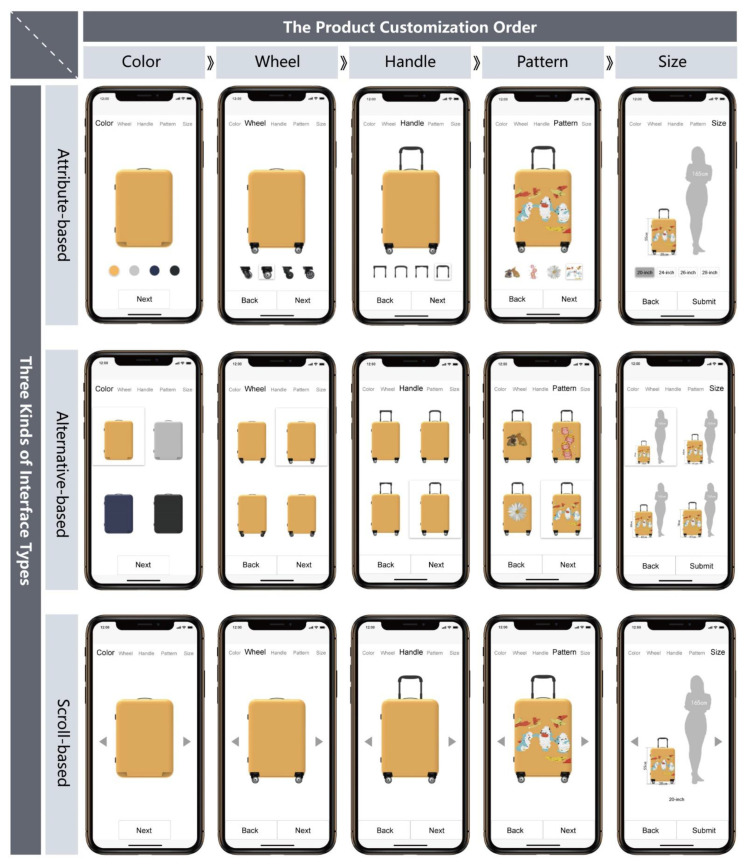
Three different interface designs and product customization steps adopted in the experiment.

The spaces for attributes and navigation on different interfaces are all the same in interface design. The text at the space for attributes is arranged horizontally, and for the attributes being customized currently, the text is in large black font, while for others, the text is in gray small font. The spaces for products are different in design for the three interfaces (see Figure 4). Taking the color selection in Step 1 for instance, the attribute-based interface arranges the color scheme horizontally just below suitcase, and the users may click the colors of suitcases for selection, the alternative-based interface displays all optional colors of suitcases simultaneously and scroll-based interface switches different colors of suitcases through the left or right gray triangle button. We have also applied a projection effect on the alternative-based and attribute-based interfaces, where the selected schemes float up to alert the users.

We also conducted a pre-test of customization system and invited five experts with experience in relevant interaction design to perform a Heuristic Evaluation on the interfaces. According to the experts’ opinions, we modified some of the design details in visual interaction and confirmed that the interface design conformed to the current business practices and expressions. Through communication with manufacturers of suitcases, we confirmed that all customization options could be finally delivered to the users through the cooperation of upstream and downstream supply chains.

### 3.3. Experimental Procedure

The tests were performed in the restroom of local labor market and a classroom of the university, so as to provide a relatively quiet experimental environment for the participants. The 72 subjects were divided into three groups, 12 college students and 12 industrial workers each group. For each group, one of three interfaces (i.e., scroll-based, alternative-based, and attribute-based interfaces) was adopted. The experiment process was divided into three stages. Stage 1 was for introduction, i.e., an assistant introduced the experiment, and how to operate the interfaces as well, and answered the subjects’ questions. Stage 2 was for task operation. At this stage, the subjects conducted online customization of suitcases. An iPhone 11 Pro Max equipped with the customization system was provided for each group, and each subject used it to complete the suitcase customization task. Each subject should complete the task within five minutes, and an assistant used a stopwatch timer to record the completion time for the task. Stage 3 was for the user experience assessment. After the suitcase customization task, the subjects completed the System Usability Scale (SUS) and NASA-TLX task load scale. The subjects were also asked to complete a questionnaire, which were developed by Park et al. [47] was specifically for mobile devices like smartphone and used for user value evaluation based on the 5 elements (self-satisfaction, pleasure, sociability, customer need, and attachment). User value was reported to be one of the most important elements that influence user experience [47], and we want to know whether the user value is correlated with the dependent variables in this study. We conducted a brief interview with each subject to further understand their psychological feelings in the process using the interfaces.

The content of the experiment task requires the participants to use the prototype system to complete the customization of suitcases based on their own preferences. No matter which interface is assigned to a subject, the product customization order is always as follows: color, wheel, handle, pattern, size, which contain almost all the details of the suitcases in current market for customers to choose. Once a suitcase attribute is selected, the subject may modify the previous attribute through the “Back” button at the bottom of the interface or click the “Next” button to select the next attribute. Prototype system adopts an “incremental custom” mode, i.e., each attribute was selected based on the previous one (For example, as choosing wheels, if a user selected yellow for previous color attribute, the interface will have different wheel styles of yellow suitcases for the user to choose). Figure 4 showed how each step in the customization process appeared on different interfaces. After the last attribute is selected, the subject may click “Submit” to submit the customization plan, and then “Thank you for customization” will appear on the interface, indicating that the customization process is over.

## 4. Results

This experiment adopted a 2 (educational background) ×3 (interface type) between-participants design, and the dependent variables were evaluations of task performance, system usability and task load. As the dependent variables were approximately normally distributed and passed the test for homogeneity of variances, we utilized the Two-Way ANOVA for analyzing relevant experimental data with IBM SPSS (version 24). For significantly different factors, a post hoc test was conducted.

### 4.1. Task Performance Analysis

To understand whether there is difference in task performance between interface types and educational backgrounds, the participants were asked to select five attribute options for suitcases (color, wheel, handle, pattern, and size), after completion of each selection, the participants could click “Next” button on the interface for another selection, or “back” button on the interface for modifying the previous decisions, until the participants thought that all attributes meet their requirements. We obtained the total time spent by the subjects for customizing suitcases, i.e., the sum of times spent by the subjects for each of the five attributes as selecting suitcases. For the descriptive statistics and Two-Way ANOVA results concerning the mean time for task performance, see Table 1.

According to Table 1, in task performance, there were significant differences between interface types (F = 4.963, *p* = 0.010 < 0.05), indicating that there were differences when the participants operated different customization interfaces, but there was no significant difference between educational backgrounds. The interaction effects between educational backgrounds and interface types were not significant. The post hoc test showed that in terms of interface types, the alternative-based customization time (M = 2.625, Sd = 0.443) was the shortest, and significantly shorter than attribute-based customization time (M = 2.945, Sd = 0.418) and scroll-based customization time (M = 3.000, Sd = 0.512) customization time. Obviously, the alternative-based interface achieved the highest efficiency and the best effect among the three types. The reason for this is that in the alternative-based interface, the participants can intuitively see all the customization scenarios visually, and they do not need to select manually, which is undoubtedly the most efficient as only one attribute is customized at a time and the optional attributes are limited. There was no significant difference in task performance between attribute-based and scroll-based interface types because both types required participants to manually select attribute values.

### 4.2. System Usability Evaluation

After the customization task was completed, participants were asked to score by System Usability Scale (SUS), a five-point scale consisting of 10 questions. The higher the score, the higher the subject’s evaluation on usability of the system. In addition to obtaining total score, SUS can also be divided into two subscales [48], the Learnability subscale composed of Term 4 and Term 10, and the Usability subscale composed of the other eight terms. This study also analyzed the two subscales.

#### 4.2.1. Evaluation on the Total SUS Score

According to descriptive statistics and two-way ANOVA in Table 2, educational background had no significant influence on the total SUS score (F = 2.182, *p* = 0.144 > 0.05), and interface type had no significant influence on the total SUS score (F = 0.587, *p* = 0.559 > 0.05). Although there was no significant difference between educational background and interface type, there was an interaction between educational background and interface type (F = 5.130, *p* = 0.008 < 0.05).

**Figure 5:**
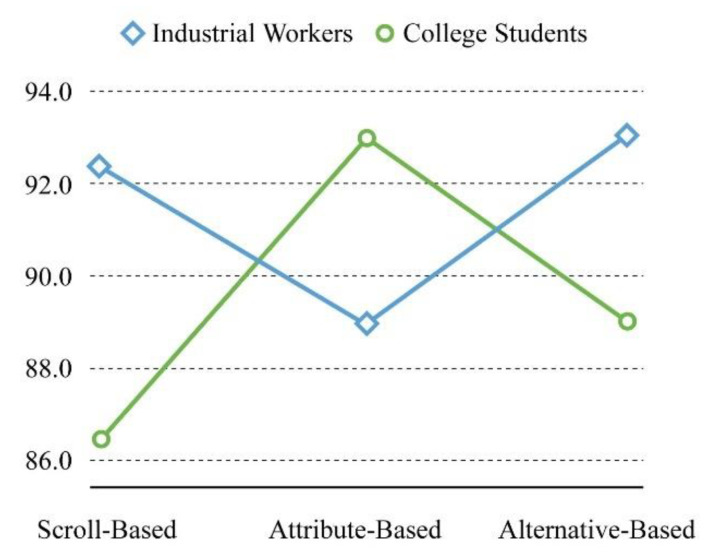
The interaction diagram regarding total SUS score.

For the interaction of total SUS score, see Figure 5. The system usability score among industrial workers was higher than that among college students for the scroll-based (M = 92.292, Sd = 5.883) and alternative-based (M = 93.125, Sd = 5.014) interfaces. However, for the attribute-based interface, the score among college students (M = 92.917, Sd = 4.981) was higher than that among industrial workers (M = 88.958, Sd = 6.166).

#### 4.2.2. SUS Learnability Evaluation

The combined score of Term 4 and Term 10 of the SUS scale was used to evaluate the learnability of system. According to Table 3, there was no significant difference between educational backgrounds in the evaluation on interface learnability (F = 0.380, *p* = 0.540 > 0.05), or significant difference between interface types in the evaluation on interface learnability (F = 2.602, *p* = 0.082 > 0.05). There was no significant interaction between educational background and interface type for the interface learnability (F = 0.289, *p* = 0.750 > 0.05).

#### 4.2.3. SUS Usability Evaluation

The system usability was evaluated through adding the remaining eight terms of the SUS scale (see Table 4). There was no significant difference in system usability between educational backgrounds (F = 1.194, *p* = 0.278 > 0.05) and between interface types (F = 0.144, *p* = 0.866 > 0.05), but there was a significant difference in interaction (F = 4.804, *p* = 0.011 < 0.05), which indicated that in the learnability and usability subscales, system usability caused the interaction of total SUS score.

### 4.3. Task Load Evaluation

The NASA Task Load Index (NASA TLX) was developed by NASA. For the NASA TLX, Likert Scale is used for relevant evaluation at seven levels, and the scale is divided into six subscales, i.e., mental demand, physical demand, temporal demand, performance, effort, and frustration, in which the performance is of inverse proposition, i.e., the higher the score, the lower the task load, while the lower the score, the lower the task load for the other five subscales. This study was to find out whether the participants with different educational backgrounds had different task loads in various interfaces based on this.

#### 4.3.1. Mental Demand

The mental demand was used to detect the memory and thinking capacities cost by the participants as they performed the customization task. The descriptive statistics and two-way ANOVA results are presented in Table 5. In mental demand, there was no significant difference between educational backgrounds (F = 0.397, *p* = 0.531 > 0.05), but there was between interface types (F = 6.397, *p* = 0.003 < 0.05). A post hoc test showed that alternative-based mental demand (M = 3.04, Sd = 1.160) was significantly higher than the scroll-based one (M = 2.08, Sd = 0.830), which indicated that the participants cost memory and thinking capacities in the scroll-based interface lower than in the alternative-based interface.

**Figure 6:**
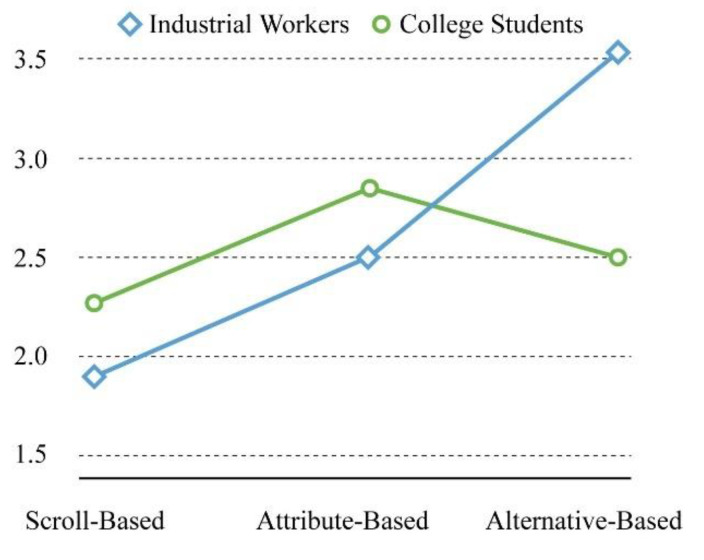
The interaction diagram regarding mental demand.

A significant interaction was observed between educational background and interface type (F = 4.587, *p* = 0.014 < 0.05), indicating that the collocation between interface type and educational background could affect the mental demand of the participants. For the interaction, according to Figure 6, in the scroll-based interface, the mental demand of college students (M = 2.25, Sd = 0.965) was higher than that of industrial workers (M = 1.92, Sd = 0.669). In the attribute-based interface, the mental demand of college students (M = 2.83, Sd = 0.937) was still higher than that of industrial workers (M = 2.50, Sd = 0.905), but in the alternative-based interface, the opposite was true, i.e., the mental demand of industrial workers (M = 3.58, Sd = 1.165) was higher than that of college students (M = 2.50, Sd = 0.905). Different from the previous post hoc test, the mental demand of college students was consistent in the two interfaces, i.e., scroll-based and alternative-based interfaces, but the performance of industrial workers was consistent with the post hoc test, that is, the scroll-based operation minimized the participants’ mental demand.

#### 4.3.2. Physical Demand

Physical demand was used to test whether the participants needed many actions or buttons to complete relevant operations as they completed the customization task. The descriptive statistics and two-way ANOVA results were as shown in Table 6. No significant difference was observed in the physical demand of participants between educational backgrounds (F = 0.017, *p* = 0.896 > 0.05); there was a significant difference in physical demand of participants between interface types (F = 7.774, *p* = 0.001 < 0.05); the interaction between educational background and interface type was not significant (F = 2.385, *p* = 0.100 > 0.05). A post hoc test found that the alternative-based physical demand (M = 2.29, Sd = 0.859) was significantly lower than the attribute-based one (M = 3.08, Sd = 0.717) and scroll-based one (M = 3.25, Sd = 1.113), which indicated that alternative-based mode required the least actions of the participants, for selection in each customization interface, the participants do not have to do it manually.

#### 4.3.3. Temporal Demand

The temporal demand was used to test whether the participants could operate the interface leisurely without pressure as they completed the customization task. The descriptive statistics and two-way ANOVA results were as shown in Table 7. There was no significant difference in the temporal demand of participants between educational backgrounds (F = 0.725, *p* = 0.397 > 0.05); there was no significant difference in the temporal demand of participants between interface types (F = 0.844, *p* = 0.435 > 0.05), or the interaction between educational background and interface type was not significant (F = 0.903, *p* = 0.410 > 0.05).

#### 4.3.4. Effort

The effort was used to test whether the participants have to work very hard to learn how to operate the system as they complete the customization task. According to the descriptive statistics and two-way ANOVA results (see Table 8), there was a significant difference in the effort of participants between educational backgrounds (F = 10.861, *p* = 0.002 < 0.05), and there was a significant difference in the effort of participants between interface types (F = 3.962, *p* = 0.024 < 0.05). A post hoc test showed that the scroll-based effort (M = 2.29, Sd = 1.042) was significantly lower than the alternative-based one (M = 2.96, Sd = 0.908), which indicated that the scroll-based interface was more acceptable, and the learning cost for the scroll-based interface was lower than that for attribute-based and alternative-based interfaces.

**Figure 7:**
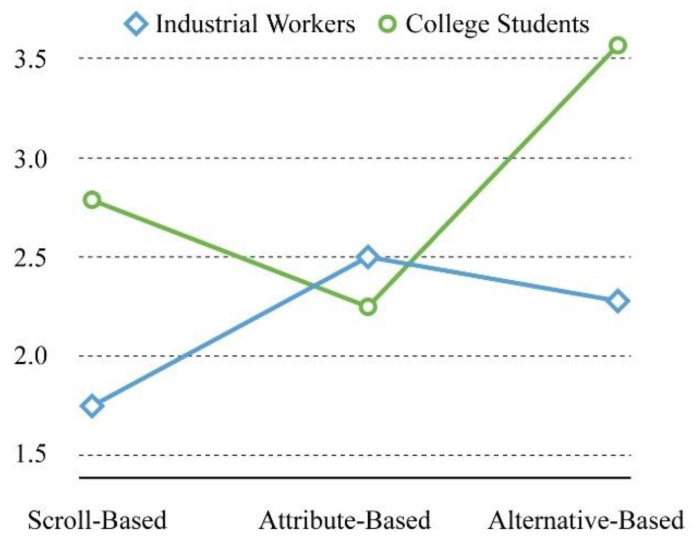
The interaction diagram regarding effort.

There was a significant interaction between educational background and interface type (F = 5.074, *p* = 0.009 < 0.05), indicating that the combination of the two factors could affect the participants’ feelings of effort. For the interaction, according to Figure 7, in the scroll-based interface, the effort of industrial workers (M = 1.75, Sd = 0.754) was lower than that of college students (M = 2.83, Sd = 1.030). In the alternative-based interface, the effort of industrial workers (M = 2.33, Sd = 0.492) was still lower than that of college students (M = 3.58, Sd = 0.793), but in the attribute-based interface, the opposite was true, i.e., the effort of the college students (M = 2.25, Sd = 1.055) was lower than the industrial workers (M = 2.50, Sd = 1.087), and the industrial workers need higher learning cost for the system operation mode.

#### 4.3.5. Performance

The performance was used to test the participants’ satisfaction during the customization process. The performance is of negative proposition, i.e., the higher the score, the higher the satisfaction. According to Table 9, there was no significant difference in the self-evaluation of participants on their performance between educational backgrounds (F = 0.159, *p* = 0.691 > 0.05); there was also no significant difference in the self-evaluation of participants on their performance between interface types (F = 2.242, *p* = 0.114 > 0.05), or the interaction between educational background and interface type was not significant (F = 0.053, *p* = 0.948 > 0.05).

#### 4.3.6. Frustration

Frustration was used to test how much frustration the participants feel when they perform the customization task. According to Table 10, there was a significant difference in the frustration of participants between educational backgrounds (F = 11.374, *p* = 0.001 < 0.05); there was no significant difference in the frustration of participants between interface types (F = 3.011, *p* = 0.056 > 0.05), or the interaction between educational background and interface type was not significant (F = 2.294, *p* = 0.109 > 0.05). In general, the frustration among industrial workers (M = 2.72, Sd = 1.059) was higher than among college students (M = 1.97, Sd = 0.910) in the customization process.

### 4.4. Correlation Analysis

We asked subjects to complete a questionnaire designed by Park et al. [47], so as to understand the correlation between the user value of mobile devices and the dependent variables. In this questionnaire, there are 16 questions used for evaluating the five elements of user value (self-satisfaction, pleasure, sociability, customer need, and attachment). Each element of user value can be measured with magnitude estimation using a Likert scale (from one “strongly agree” to five “strongly disagree”). For example, the sum of scores for the three questionnaire items is the total score of “customer need”: (1) My phone seems to be useful because there are many things I can do with it; (2) I am satisfied with that I can download applications that I want to have; (3) I can obtain the information that I want through the mobile phone. However, we did not find any element score in the user value with statistically significant correlation with task performance, system usability and task load scores of users (*p* > 0.05).

The study also assessed the correlation between task performance, system usability, and task load, and found that the system usability score had a significantly negative correlation with the effort in task load (*p* = 0.017 < 0.05), with Pearson correlation coefficient −0.280 (see Table 11), which indicated that the higher the system usability, the lower the participants’ effort in the process of customization.

## 5. Discussion

When an information presentation form matches a task correctly, the task performance will become faster and better. This is an important point of the theory of cognitive fit [5]. This study aims to explore the differences in task performance, system usability, and operating load between different interface types, and whether educational background has a relevant influence.

### 5.1. Interface Type

Different interface designs did lead to significant differences in task performance. Alternative-based interface enabled the participants to complete the customization tasks in the shortest time, while attribute-based and scroll-based interfaces cost the participants more time. The reason may be that in the alternative-based interface, the participants can intuitively see all the customization scenarios visually, and they do not need to select relevant properties manually. In a previous study, Kamis et al. [16] emphasized the alternative-based interface would bring people a relatively high mental demand, but they could complete customization of all product attributes in a single page. The study broke various attributes for customization into stages and switched the customization pages by “Back” and “Next”. So, the users only had to complete customization task for one attribute at a time, thus effectively reducing the cognitive complexity of interfaces, and promoting the efficiency of alternative-based interface design. Another possible reason is that users’ visual attention seems to be relatively high in an interface with high cognitive load [49], and the high attention may accelerate the speed for customization task completion.

In task performance, there was no significant difference between attribute-based and scroll-based interface designs. The reason might be that the customization task was too simple for the participants to make a substantial difference in operating time. Some other investigators put forward such an explanation [5], but the Bacic and Henry [41] pointed out that although there was no significant difference in task performance among the participants, there might be a difference in psychological feeling and cognitive ability, i.e., some system designs made people feel more “tough”, and this difference was often caused by the users’ individual characteristics. This conclusion was still true for the task load test results of this study.

There was a significant difference in mental demand between interface types, the alternative-based mental demand was significantly higher than the scroll-based one, which again confirmed the conclusion of Kamis et al. [16]. Alternative-based interface made the participants intuitively see all the possible customization solutions visually. For this, Dellaert and Stremersch [50] pointed out that when the users had a large number of options, there might be a “paradox of choice”, which may cause frustration. Piller and Tseng [51] also thought that excessive alternatives reduce their subjective value to users, which in turn leads to relevant decisions delayed or such tasks deemed difficult. However, the above conclusion is different from Randall et al. [52], who believed that an interface that displayed both the current and previous configurations made it easy for users to compare the differences between customization attributes, and all design parameters and product attributes should be displayed.

As for physical demand, there was significant physiological difference among user for different interfaces. Specifically, the users’ physical demand in the attribute-based and scroll-based interfaces was higher than in the alternative-based interface. This is easy to explain: In the alternative-based interface, consumers can directly select their desired customization solutions without need to manually select the attribute style, while in the scroll-based and attribute based interfaces, the consumers need to complete the customization tasks through continuously swiping or clicking the screen.

Effort reflects whether the users need to study hard to use the system freely. The test results showed that there were significant differences between interface types. A post hoc test showed that the scroll-based effort was significantly lower than the alternative-based one. According to Yu and Kong [23], when users are driven by tasks, they tend to use a simple and intuitive interface to help them complete the tasks, and at this point, what the users care is not how smartphones operate or navigate, but whether the interactive features are conductive to their goals. The test results showed that the scroll-based interface design was considered the simplest by the users.

### 5.2. Educational Background

The influence on dependent variables of education background difference is less extensive than of interface difference. The influence exists mainly in effort and dependency. Interestingly, industrial workers exerted less effort as using the customization system but tended to feel frustrated. On the contrary, college students exerted more effort as operating the system, but seldom felt frustrated, which again confirmed the opinion of Bacic [41], who reckoned that the score of task performance could not fully represent the psychological feelings of the subjects. In this experiment, however, there was no difference in task performance of operating interfaces between college students and industrial workers due to their education background, but their psychological feelings at the effort and frustration levels were significantly different.

In the interviews, some of the workers said that the online customization of suitcases provided them with many options, which were stressful for them, for they were not in line with their daily consumption habits. Although some workers from the rural areas had a high income in cities, they had to bear additional expenses (e.g., for rent, transportation, etc.), and many of them had to support their family members in rural areas, which limited their consumption capacity, and made them tend to select cheap suitcases. As far as we know, the richness in suitcase style depends on the cost. As the workers are used to the simple suitcases of low price, they will be overwhelmed by the variety of options offered by the customization system and feel frustrated.

Another possible reason may be that the cognitive structure and verbal sequential memory are underdeveloped among the less educated [53], but we also believe that the self-efficacy with computers and self-service technology may be influenced by previous experience and repeated training in related technologies [54,55,56]. Given that all of the participants were using the system for the first time, if the industrial workers were given more operation opportunities, their self-efficacy could be effectively improved and their frustration level could be reduced. The college students are not faced with the same financial pressure as industrial workers because Chinese parents pay for their children’s college expenses generally. More importantly, as Gen Z consumers, they have been accustomed to online shopping and even become dependent on it. Online shopping platforms need no space for shelves as providing products various than brick-and-mortar stores, and therefore, the college students are more likely to accept the suitcase customization system with abundant combinations of styles, which may be the reason why college students are less frustrated than workers. Then, why did the student exert more effort to the customization system as compared with the workers? Some college students said that they liked the rich options offered by the customization system, but it was not easy to obtain an optimal style of suitcase, and so, they had to click the “Back” button to change the previous options, which made them exert more effort.

### 5.3. Interaction between the Variables

In terms of the overall evaluation on system usability, there was an interaction between educational background and interface type, which was caused by the influence of subscale usability. The collocation of educational background and interface type may affect the users’ evaluation on the system usability. The interaction plot showed that the college students had a high score for the attribute-based interface design, while the industrial workers preferred scroll-based and alternative-based interface designs, i.e., the industrial workers believed that the scroll-based and alternative-based interfaces were relatively easy to learn, and they felt confident in system use. There are two possible explanations for such a result. First, for the users with relatively low education, it is difficult to understand the hierarchical information structure of the system [28]. In the three kinds of interface design, the information architecture for scroll-based and alternative-based designs is relatively simple, while that for attribute-based design involves two layers, namely the attribute layer and the product display layer. Second, the participants with relatively high education level are less affected by interface background and dynamic effects, while participants with low education level are more interested in design elements that can affect their senses [32]. Obviously, scroll-based visual design is more dynamic, and so, the industrial workers prefer the design. According to the feedback from the interview in post hoc test, industrial workers generally reckoned that the left and right scroll-based interface was relatively interesting and easy to learn in operation, and they could master the system operation skills without extra studies.

Among the seven indicators for evaluating task load, educational background and interface type interacts for scores at the mental demand and effort levels. In terms of mental demand, on the scroll-based and the attribute-based interfaces, the mental demand of industrial workers was lower than that of college students, which indicated that these two interface designs were more conducive to the information processing ability of workers performing online customization tasks. On the alternative-based interface, the workers had a high mental load, which suggested that such an interface increased the workers’ mental load because they had to receive complex product information in a limited time. However, the mental load is not an absolute standard for evaluation of interface design, i.e., the lowest mental load is not equivalent to the best interface design. In fact, a moderate mental load makes people under the best working state, and excessively low mental load makes people distracted or even dulls their responses; while excessively high mental load makes people fatigue, for which they may become irritable, distressed and etc. [57]. Therefore, it is important to balance, i.e., make the users have appropriate mental load as operating the interfaces as avoiding excessively high or low mental stress. According to the average score of mental load in different interface types, the attribute-based interface is between scroll-based and alternative-based interfaces for mental load. However, whether attribute-based interface design creates the most beneficial moderate mental load for users is to be studied in the future.

On the scroll-based and alternative-based interfaces, the industrial workers exerted less effort as compared with the college students, but they exerted even more effort on the attribute-based interface, which indicated that the scroll-based and alternative-based interfaces allow workers to exert less effort for comparing the configurations of suitcases and making a decision. We explained the interaction in terms of the relationship between the effort and the progression rate of a goal. A previous study has shown that when the achievement level of a goal is low, a rapid progression rate indicates a high expectation for achieving the goal, which motivates people to exert more effort for achieving the goal. When the achievement level of the goal is high, people pay more attention to when the goal can be achieved. At this point, a low progression rate makes people exert more effort to achieve the goal [58]. In other words, dependent on the achievement level the goal, both high and low progression rates make people exert more effort.

In this study, completing the suitcase customization task is the subjects’ definite goal, a simple goal in our opinion. Considering that only five attributes for selection, i.e., when the subjects begin to select the first attribute, the achievement level for the goal has been high. At this point, people care more about when the customization task will be completed, and so, long customization time makes the subjects exert more effort. We reviewed the customization time spent by the workers on different interface types (see Table 1) and found that the customization time on the attribute-based interface (M = 3.183) was longer than that on the scroll-based interface (M = 2.975) and alternative-based interface (M = 2.775), and on the attribute-based interface, the slow progression rate may have resulted in more effort exerted by the workers. Interestingly, the college students exerted the most effort on the alternative-based interface. Considering that the customization time on the alternative-based interface (M = 2.475) was shorter than on the scroll-based interface (M = 3.025) and on the attribute-based interface (M = 2.792), i.e., high progression rate made the college students exert more effort, which was contrary to the result of the industrial workers, the college students reckoned that the achievement level of the goal at the beginning of customization was relatively low. Maybe this confirmed our analysis in the previous section, i.e., the college students had relatively high requirements for the suitcase customization scheme. In order to satisfy themselves, they clicked the "Back" button to modify the previous options, which make them think that there is still a long way to go for completing the customization task.

## 6. Conclusions

This study explored the influence of different interface types and educational backgrounds on task performance and subjective evaluation of mobile customization systems. A structured interview showed that the participants’ overall evaluation on the interfaces was satisfactory, and all of them could complete the customization work independently. The main conclusions are as follows: (1) The customization systems using different interface types led to significant differences in task performance. The alternative-based interface design achieved the best results in task performance, while there was no significant difference in task performance between attribute-based and scroll-based interfaces. (2) The collocation of educational background and interface type may affect the users’ evaluation on the system usability. The industrial workers thought that the scroll-based and alternative-based interfaces were more usable than the other one, while the college students preferred the attribute-based interface. (3) There was a significant difference in task load between interface types. The scroll-based interface gave the users the lowest mental demand, while alternative-based interface gave the users the lowest feeling of physical demand but cost more effort. (4) There was a significant difference in user task load between educational backgrounds. The industrial workers showed relatively low effort in the scroll-based and alternative-based interfaces, while the college students showed relatively low effort in the attribute-based interface. (5) A correlation analysis showed that there was a significant negative correlation between system usability score and effort in task load.

As with most studies, our study has some limitations. First, the number of participants may limit our ability to identify and expand study findings broadly. Second, in order to control unnecessary factors, we selected a relatively quiet site for testing, and so, the applicability of our study results in complex scenes (e.g., in a noisy environment, and walk while playing) is to be verified. At last, price was not taken into account in the interface design, i.e., when customizing each attribute, the price was not displayed below the attribute value. For some attributes (e.g., color), the price was consistent, but for some attributes (e.g., wheel and size), the price was not consistent. The correlation analysis showed that the user value of mobile devices was irrelevant to the dependent variables in this study. However, this does not mean that the influence of user value on the user experience for the customization system will not be considered, for the concept of customization system is a very broad. In future studies, we will be very interested in using the five elements (self-satisfaction, pleasure, sociability, customer need, and attachment) constituting the user value as independent variables to study the availability of other customization systems. We hope that our study may stimulate constructive debate about the cognitive processes of individualized customization.

## Figures and Tables

**Table 1:** Descriptive statistics and two-way ANOVA on task performance (unit: minute).

Task Performance	Industrial Workers		College Students		M		EB	IT		EB × IT
	M	SD	M	SD	M	SD	*p*	*p*	Post hoc	*p*
A	2.975	0.412	3.025	0.614	3.000	0.512	0.075	**0.010**	(A, B) > C	0.279
B	3.117	0.497	2.792	0.247	2.954	0.148				
C	2.775	0.283	2.475	0.529	2.625	0.443				
M	2.956	0.420	2.764	0.527						

A = scroll-based, B = attribute-based, C = alternative-based, EB = educational background, IT = interface type.

**Table 2:** Descriptive statistics and two-way ANOVA on the total System Usability Scale (SUS) score.

Total SUS Score	Industrial Workers		College Students		M		EB	IT	EB × IT
	M	SD	M	SD	M	SD	*p*	*p*	*p*
A	92.292	5.883	86.667	4.687	89.479	5.943	0.114	0.559	**0.008**
B	88.958	6.166	92.917	4.981	90.938	5.843			
C	93.125	5.014	88.958	6.524	91.042	6.076			
M	91.458	5.836	89.514	5.911					

A = scroll-based, B = attribute-based, C = alternative-based, EB = educational background, IT = interface type.

**Table 3:** Descriptive statistics and two-way ANOVA of SUS learnability.

SUS Learnability	Industrial Workers		College Students		M		EB	IT	EB × IT
	M	SD	M	SD	M	SD	*p*	*p*	*p*
A	15.833	1.946	15.417	2.344	15.625	2.118	0.540	0.082	0.750
B	16.667	2.462	15.833	2.219	16.250	2.331			
C	17.083	2.984	17.292	2.251	17.188	2.587			
M	16.528	2.484	16.181	2.352					

A = scroll-based, B = attribute-based, C = alternative-based, EB = educational background, IT = interface type.

**Table 4:** Descriptive statistics and two-way ANOVA of SUS usability.

SUS Usability	Industrial Workers		College Students		M		EB	IT	EB × IT
	M	SD	M	SD	M	SD	*p*	*p*	*p*
A	76.458	6.437	71.250	5.057	73.854	6.255	0.278	0.866	**0.011**
B	72.292	8.010	77.083	4.747	74.688	6.889			
C	76.042	6.781	71.667	5.573	73.854	6.468			
M	74.931	7.159	73.333	5.670					

A = scroll-based, B = attribute-based, C = alternative-based, EB = educational background, IT = interface type.

**Table 5:** Descriptive statistics and two-way ANOVA on mental demand by NASA-Task Load Index (TLX).

Mental Demand	Industrial Workers		College Students		M		EB	IT		EB × IT
	M	SD	M	SD	M	SD	*p*	*p*	Post hoc	*p*
A	1.92	0.669	2.25	0.965	2.08	0.830	0.531	**0.003**	A < C	**0.014**
B	2.50	0.905	2.83	0.937	2.67	0.917				
C	3.58	1.165	2.50	0.905	3.04	1.160				
M	2.67	1.146	2.53	0.941						

A = scroll-based, B = attribute-based, C = alternative-based, EB = educational background, IT = interface type.

**Table 6:** Descriptive statistics and two-way ANOVA on the physical demand by NASA-TLX.

Physical Demand	Industrial Workers		College Students		M		EB	IT		EB × IT
	M	SD	M	SD	M	SD	*p*	*p*	Post hoc	*p*
A	2.92	0.996	3.58	1.165	3.25	1.113	0.896	**0.001**	(A, B) > C	0.100
B	3.17	0.835	3.00	0.603	3.08	0.717				
C	2.50	0.674	2.08	0.996	2.29	0.859				
M	2.86	0.867	2.89	1.116						

A = scroll-based, B = attribute-based, C = alternative-based, EB = educational background, IT = interface type.

**Table 7:** Descriptive statistics and two-way ANOVA on the temporal demand by NASA-TLX.

Temporal Demand	Industrial Workers		College Students		M		EB	IT	EB × IT
	M	SD	M	SD	M	SD	*p*	*p*	*p*
A	3.00	0.739	2.83	1.030	2.92	0.881	0.397	0.435	0.410
B	2.58	0.793	3.17	1.030	2.88	0.947			
C	2.50	0.522	2.67	1.435	2.58	1.060			
M	2.69	0.710	2.89	1.166					

A = scroll-based, B = attribute-based, C = alternative-based, EB = educational background, IT = interface type.

**Table 8:** Descriptive statistics and two-way ANOVA on the effort by NASA-TLX.

Effort	Industrial Workers		College Students		Mean		EB	IT		EB × IT
	M	SD	M	SD	M	SD	*p*	*p*	Post hoc	*p*
A	1.75	0.754	2.83	1.030	2.29	1.042	**0.002**	**0.024**	A < C	**0.009**
B	2.50	1.087	2.25	1.055	2.38	1.056				
C	2.33	0.492	3.58	0.793	2.96	0.908				
M	2.19	0.856	2.89	1.090						

A = scroll-based, B = attribute-based, C = alternative-based, EB = educational background, IT = interface type.

**Table 9:** Descriptive statistics and two-way ANOVA on performance by NASA-TLX.

Performance	Industrial Workers		College Students		M		EB	IT	EB × IT
	M	SD	M	SD	M	SD	*p*	*p*	*p*
A	5.83	0.718	5.83	0.835	5.83	0.761	0.691	0.114	0.948
B	5.25	0.754	5.33	0.778	5.29	0.751			
C	5.50	1.000	5.67	1.155	5.58	1.060			
M	5.53	0.845	5.61	0.934					

A = scroll-based, B = attribute-based, C = alternative-based, EB = educational background, IT = interface type.

**Table 10:** Descriptive statistics and two-way ANOVA on frustration by NASA-TLX.

Frustration Level	Industrial Workers		College Students		M		EB	IT	EB × IT
	M	SD	M	SD	M	SD	*p*	*p*	*p*
A	2.92	0.996	2.42	0.793	2.67	0.917	**0.001**	0.056	0.109
B	2.17	1.115	1.83	0.937	2.00	1.022			
C	3.08	0.900	1.67	0.888	2.38	1.135			
M	2.72	1.059	1.97	0.910					

A = scroll-based, B = attribute-based, C = alternative-based, EB = educational background, IT = interface type.

**Table 11:** Correlation analysis on system usability and effort.

Correlation Analysis		SUS (Total Score)	Effort
SUS (total score)	Pearson correlation	1	−0.280
	*p*		**0.017**
	N	72	72
Effort	Pearson correlation	−0.280	1
	*p*	**0.017**	
	N	72	72

## Data Availability

Not applicable.
